# Perceived Preparedness of Health Care Students for Providing Cardiovascular Disease Risk Assessment and Management

**DOI:** 10.3390/pharmacy5010009

**Published:** 2017-02-21

**Authors:** Monica Zolezzi, Oraib Abdallah, Suad Aden, Stella Major, Diana White, Alla El-Awaisi

**Affiliations:** 1 College of Pharmacy, Qatar University, P.O. Box 2713, Doha, Qatar; oa095387@student.qu.edu.qa (O.A.); suad.aden2014@gmail.com (S.A.); elawaisi@qu.edu.qa (A.E.); 2 Weill Cornell Medicine—Qatar, Qatar Foundation, P.O. Box 24144, Doha, Qatar; scm2009@qatar-med.cornell.edu; 3 University of Calgary Qatar, P.O. Box 23133, Doha, Qatar; dlwhite@ucalgary.edu.qa

**Keywords:** cardiovascular disease, risk assessment, pharmacy, medical, nursing, students, education, interprofessional

## Abstract

Early assessment and management of risk factors is known to have significant impact in preventing cardiovascular disease (CVD) and its associated burden. Cardiovascular disease risk assessment and management (CVDRAM) is best approached by teamwork across health care professionals. This study aimed at assessing health care students’ (HCSs) knowledge about the parameters needed for estimating CVD risk, their self-assessed preparedness/confidence and perceived barriers for the provision of CVDRAM services through a survey administered to third and fourth year pharmacy, medical, and nursing students in Qatar. Although all student cohorts achieved similar knowledge scores, less than half (*n* = 38, 47%) were able to identify all of the six main risk factors necessary to estimate absolute CVD risk, and a third (32%) were unable to identify total cholesterol as an independent risk factor necessary to estimate CVD risk. Training on the use of CVD risk assessment tools differed among the three student cohorts. All student cohorts also perceived similar levels of preparedness in CVDRAM. However, pharmacy students reported the highest preparedness/confidence with the use of the latest CVDRAM guidelines. The majority of statements listed under the barriers scale were perceived by the students as being moderate (median score = 3). Poor public acceptance or unawareness of importance of estimating CVD risk was the only barrier perceived as a major by nursing students. Future integration of interprofessional educational (IPE) activities in the CVDRAM curricula of HCSs may be a suitable strategy to minimize barriers and foster collaborative practice for the provision of CVDRAM services in Qatar.

## 1. Introduction

According to the World Health Organization (WHO), cardiovascular diseases (CVDs) are associated with high rates of mortality and morbidity. In 2012, CVDs contributed to 31% of deaths worldwide [1]. Several risk factors have been confirmed as major independent predictors of CVD, with their modification reducing the risk of major cardiovascular events in people with diagnosed or undiagnosed CVD [2,3,4]. Studies have shown that absolute (global/total) risk assessment is essential as multiple risk factors confer greater risk than the sum of their components [5,6]. WHO recognizes that an important aspect of decreasing the global burden derived from CVDs is to shift the focus from treatment to prevention. WHO also recommends that a paradigm shift is required from treatment of CVD risk factors in isolation to comprehensive cardiovascular risk assessment and management (CVDRAM) [7].

In Qatar, CVDs accounted for 24% of the total deaths reported in 2014 [8]. Local studies have also reported high prevalence rates of CVD risk factors, such as hyperlipidemia, obesity, hypertension, and diabetes [9]. In the 2014 Qatar Health Report, the Supreme Council of Health indicated that Qatar has non-communicable disease rates akin to those of aging countries despite the share of people aged 65+ being the world’s second lowest [10]. This is largely driven by sedentary lifestyles which necessitates increased prevention at all levels of care. Thus, implementing CVDRAM services would align well with Qatar’s health care reform which aims at remodeling services to focus on primary prevention.

Despite the potential usefulness of CVDRAM, several barriers for its routine adoption in primary care settings have been identified, such as a lack of understanding on the part of health care professionals (HCPs) of absolute CVD risk estimation, HCPs time constraints to perform CVD risk assessments, and the fact that individuals without disease symptoms may not have routine contact with HCPs to have their CVD risk assessed [11,12]. Interdisciplinary approaches have been recommended to improve the success of CVD risk factor assessment and modification [12,13,14,15]. Interdisciplinary teams delivering effective CVDRAM have been reported in the literature. Sandhoff and colleagues [16] described a collaborative cardiac care service (CCCS) which demonstrated improvement in cholesterol screenings and in the proportion of patients achieving target values of modifiable risk factors. Reductions in all-cause mortality associated with CVD in the patients followed by the service, and high satisfaction with the CCCS, was reported by both patients and physicians. Similar positive outcomes were demonstrated in other team care approaches [17,18,19]. As such, adequately preparing health care students (HCSs) for the provision of interprofessional CVDRAM services may be a valuable strategy not only to promote the adoption and the full integration of CVD risk assessment into primary care services but, most importantly, to improve the cardiovascular health of the public. 

The aim of this study was to gain understanding of the overall preparedness of medical, nursing and pharmacy students’ for performing CVD risk assessments. Specifically, this study was developed to answer four main research questions: (1) are there any differences among these HCSs in the knowledge about the parameters needed for estimating CVD risk? (2) How prepared do these HCSs perceive themselves to be for the provision of CVDRAM services? (3) Are there any relationships between these HCSs’ demographic characteristics and their knowledge and perceived preparedness in CVD risk assessment? (4) What are the perceived barriers reported by these HCSs for the provision of CVDRAM services, particularly in the Qatari health care context? We hope that a better understanding of the overall preparedness of HCSs in CVDRAM will help inform initiatives for interprofessional education (IPE) that could foster collaborative practice for the provision of future CVDRAM services in Qatar.

## 2. Materials and Methods 

### 2.1. Study Design and Participants

The study was designed as a cross-sectional survey of pharmacy, nursing, and medical students at Qatar University College of Pharmacy (QUCPH), University of Calgary in Qatar (UCQ), and Weill Cornell Medicine in Qatar (WCM-Q). Students were eligible to participate if they had covered the cardiovascular therapeutics curricula and had been through at least one clinical/practical placement. Based on this eligibility criterion, all third and fourth year students from the three study colleges were invited to participate in an anonymous, self-administered survey (N = 207). Using a validated online calculator (Raosoft_®_ (Raosoft Inc., Seattle, WA, USA)) a sample size of 135 was calculated (95% +/− 5%). In view of the different geographical locations of the three colleges in Qatar, administration of the survey was either paper-based (group-administered to pharmacy students at QUCPH) or online (for WCM-Q medical students and UCQ nursing students) using SurveyMonkey_®_ (SurveyMonkey, San Mateo, CA, USA).

### 2.2. Survey Tool

A questionnaire which assessed pharmacy students’ knowledge, perceived preparedness/confidence and perceived barriers for the provision of CVDRAM developed elsewhere [20] was used, but it was culturally adapted and modified to fit all three student cohorts in this study. The questionnaire was divided into four sections:

**Part 1**: Students’ demographic information (five items) 

**Part 2**: Students’ knowledge and training in CVD risk factors, which was divided into two sub-sections:

Students’ training on a variety of health assessments (12 items, Yes/No)

Students’ knowledge of CVD risk factors (22 items, Yes/No/Do not know)

**Part 3**: Students’ perceived preparedness and confidence in the provision of CVDRAM (13 items anchored on a five-point Likert scale where 1 = strongly disagree, 2 = disagree, 3 = neutral, 4 = agree, 5 = strongly agree).

**Part 4**: Perceived barriers for the provision of CVDRAM services (seven items anchored on a numerical scale from 0–5, where 0 = not a barrier and 5 = major barrier).

The draft questionnaire was piloted using a sample of second-year pharmacy students who had already covered the curricula on cardiovascular therapeutics. In addition, content validation was performed by all collaborators at the three study colleges who checked for language, format, relevance, clarity, and readability of the questionnaire. Necessary modifications were carried out based on the results of the pilot testing and content validation. 

The reliability of the questionnaire was tested for internal consistency by calculating Cronbach’s alpha for the scales presented in Part 3 and 4 of the questionnaire. Applying “Cronbach’s Alpha if Item Deleted” for each statement was done to determine which domain had low consistency. Cronbach’s alpha was considered acceptable if it had values of 0.70 and above. 

The study received ethics approval from Qatar University Institutional Review Board (QU-IRB) for using a modified version of the questionnaire which was initially approved for another project targeting pharmacy participants only (QU-IRB 381-E/14). Permission to administer the questionnaire to medical and nursing students was also granted by WCM-Q and UCQ, respectively. 

### 2.3. Data Analysis

IBM Statistical Package for Social Sciences (IBM SPSS Software, version 22 (IBM Corporation, North Castle, NY, USA)) was used for analyzing the collected data. Both descriptive and inferential statistics were applied as appropriate. Testing for normality using the Shapiro-Wilk test *p*-value as a numerical output and histogram as a visual output were done. Data for all scores were found to be not normally distributed (*p* < 0.05).

From a list of health assessments, only those necessary to estimate CVD risk were assessed. A knowledge score was calculated by assigning a point for identifying each risk factor necessary for estimating absolute CVD risk (i.e., age, gender, smoking status, blood pressure, total cholesterol (TC) and high-density lipoprotein (HDL) cholesterol) with a maximum score of 6 and a minimum score of 0. A weighted score for the students’ training on the use of CVD risk calculators was also assigned one point if students were only trained on campus, two points if the training was entirely during clinical placements (without theoretical on campus component), three points if students were trained on campus and during clinical placements, and zero points if the student reported no training or no recollection if training was provided. Thus, the maximum possible score was 3 and the minimum possible score was 0. The differences in the students’ knowledge and training scores among the three cohorts were assessed using the Independent Sample of Kruskal-Wallis test which was followed by pairwise comparison to identify which group was driving the difference.

The median and interquartile ranges (IQR) were calculated for the students’ perceived preparedness/confidence and barriers scales. Median scores in the “Preparedness and Confidence” Likert scale was used to classify the students as “not prepared/not confident” (if the median score was < 3), “moderately prepared/relatively confident” (if the median score was = 3), and as “well prepared/confident” (if the median score was ≥ 4). The median score in the “Barriers” numerical scale was used to classify the barriers as “minor” (median score ≤ 2), “moderate” (median score = 3), and “major” (median score ≥ 4). The independent sample for the Kruskal-Wallis test was used for measuring the differences in responses among the three cohorts, followed by pairwise comparison.

The level of significance was set a priori at *p* ≤ 0.05. Pairwise comparisons were performed whenever there was a statistically significant difference in the scores amongst the three student cohorts. Bonferroni correction was performed to adjust the *p* value for items that reached significance. Frequency distributions were used to describe the students’ demographic information. Spearman rank-order (Rho) was used to test if any student demographics or if the students’ perceived preparedness/confidence scores correlated with the students’ CVD risk assessment knowledge and training scores.

## 3. Results

### 3.1. Students’ Demographic Characteristics

Of the 207 eligible students who were invited to participate (46 pharmacy, 61 nursing. and 100 medical) only 81 students (39%) completed the questionnaire. As shown in Table 1, the majority were pharmacy students (*n* = 35, 43%), followed by medical (*n* = 25, 30.8%) and nursing (*n* = 21, 26%) students, and most were females (*n* = 67, 82.7%). More than one-third of the participants (*n* = 30, 37%) had been through more than six months of clinical placements. The majority of students (*n* = 54, 66%) were not interested in working in primary care settings. More details regarding the demographic and educational characteristics of respondents are provided in Table 1.

### 3.2. Students’ Knowledge on CVD Risk Factors for Estimating Absolute CVD Risk

Almost half of all participating students (*n* = 38, 47%) were able to identify all the main six risk factors necessary to estimate absolute CVD risk (i.e., age, current smoking status, blood pressure, gender, TC, and HDL level). The median knowledge score for all the respondent students was 5 (IQR = 1). The risk factor required to estimate absolute CVD risk that was least likely to be identified was TC (*n* = 26, 32%). As presented in Table 2, Kruskal-Wallis results suggests that there was no statistically significant difference in the mean ranks for each of the three student cohorts able to identify all the main six risk factors necessary to estimate absolute CVD risk. More nursing students (57%) than pharmacy (49%) or medical (36%) students achieved the maximum score of six, although this was not statistically significant (*p* = 0.29).

The proportion of students able to identify gender and TC as factors required for estimating absolute CVD risk was significantly different among the three cohorts (*p* = 0.030 and *p* = 0.003, respectively). The independent sample of Kruskal-Wallis, followed by pairwise comparisons, indicated that medical students (60%) were significantly less likely than nursing students (71.4%) to identify TC as necessary for estimating absolute CVD risk (*p* = 0.002). Likewise, pharmacy students (82.8%) were significantly less likely than medical students (96%) to identify gender as necessary for estimating absolute CVD risk (*p* = 0.014). Spearman’s Rho test showed no significant correlation between the students’ CVD risk factors knowledge score and certain students’ characteristics, such as academic year and total time spent in clinical placements. 

### 3.3. Students’ Training on the Use of CVD Risk Calculators

**Figure 1:**
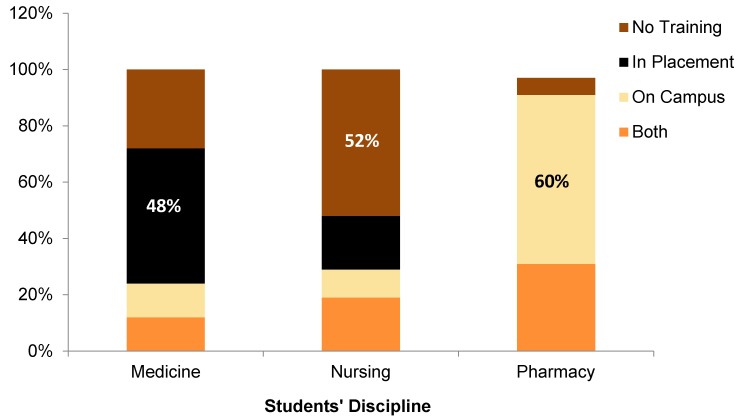
Sites for students’ training on the use of cardiovascular disease risk calculators.

The majority of students (*n* = 26, 32%) received a training score = 1 on the use of CVD risk assessment calculators, 20% received a training score = 2, and 22% received a training score = 3. Up to 26% of students received a training score = 0. There was no statistically significant difference in the training scores among the three cohorts of students (*p* = 0.197). As shown in Figure 1, about 48% of medical students received training on CVD risk calculators during their clinical rotations, whereas it was mostly in a classroom setting for pharmacy students (60%). More than half of the nursing students (52%) reported no training on CVD risk calculators. Spearman’s Rho (rs) test did not indicate a significant correlation between students’ training scores on the use of CVD risk calculators and their knowledge score on CVD risk factors required to estimate CVD risk. 

### 3.4. Students’ Perceived Preparedness and Confidence in the Provision of CVD Risk Assessment and Management

Students perceived to be well-prepared and confident for the provision of CVDRAM, as their preparedness/confidence median scores for the majority of statements tested were ≥ 4 on the Likert scale. Students were perceived to be only moderately prepared/relatively confident (median score = 3) in relation to the recommendations provided by the 2013 American College of Cardiology/American Heart Association (ACC/AHA) guidelines on CVDRAM, as well as in relation to performing physical assessments necessary to screen patients for CVD. The preparedness/confidence scores amongst the three student cohorts differed significantly only in the statement related to their awareness and understanding of the ACC/AHA guidelines (*p* = 0.001). Pairwise comparison showed that pharmacy students (median (IQR) = 4(1)) perceived to be significantly more prepared/confident than nursing students (median (IQR) = 2(1)) in relation to understanding the recommendations provided in the ACC/AHA guidelines (*p* < 0.001).

Spearman’s Rho (rs) test showed a positive and statistically significant correlation between the students’ training scores in the use of CVD risk calculators and their perceived preparedness/confidence scores in relation to using CVD risk calculators (rs = 0.26, *p* = 0.023), understanding the ACC/AHA guidelines (rs = 0.31, *p* = 0.006), recommending strategies to reduce CVD risk (rs = 0.28, *p* = 0.015) and recommending pharmacological treatments for CVD (rs = 0.27, *p* = 0.02). No significant correlation was found between the students’ knowledge scores on CVD risk factors required to estimate CVD risk and their perceived preparedness/confidence score. 

### 3.5. Students’ Perceived Barriers in the Provision of CVDRAM

Overall, the majority of statements listed under the barriers scale were perceived by the students as being moderate (median score = 3), with the exception of barriers: “Lack of resources”, “Lack of confidence and training”, and “CVD risk assessment is not an accepted scope of practice within my profession”, which were viewed as minor barriers (median scores of 2, 2, and 1, respectively).“Poor public acceptance or unawareness of importance of estimating CVD risk” was the only one perceived as a major barrier by nursing students. More details are shown in Table 3. 

Median scores assigned to “Lack of support” and “Poor public acceptance or unawareness of importance of estimating CVD risk” were significantly different amongst the three cohort of students (*p* = 0.01 and *p* = 0.001, respectively). Pairwise comparison revealed that pharmacy students (median (IQR) = 3(3)) assigned higher scores than medical students (median (IQR) = 2(3)) to the barrier “Lack of support” (*p* = 0.003), whereas nursing students (median (IQR) = 4(2.25)) assigned higher scores than medical and pharmacy students (median (IQR) = 2(3)) to the barrier “Poor public acceptance or unawareness of importance of estimating CVD risk” (*p* < 0.001 and *p* = 0.002, respectively).

### 3.6. Reliability of the Questionnaire Scales 

Cronbach’s alpha of the preparedness, confidence, and perceived barriers scales of CVD risk assessment and management questionnaire were 0.749, 0.915, 0.745, respectively, which shows very good internal consistency reliability for the scales. Results of “Alpha if Item Deleted” for all of the scales were lower than the final alpha value, except for three items in the whole tool that we may want to consider removing from the scale. These statements are: 

“I am aware of and understand the recommendations provided by the 2013 ACC/AHA Guidelines on CVD risk” under the preparedness scale. 

“I feel confident interviewing patients to identify the presence of CVD risk factors” under the confidence scale. 

“Lack of confidence and training in CVD risk assessment and management” under the barriers scale.

## 4. Discussion

This study evaluated the knowledge and reported training in CVD risk assessment by health care students (HCSs) in Qatar. To our knowledge, this is the first study that assessed the knowledge, training, perceived preparedness, and barriers of CVDRAM among HCSs. The results indicate that the three cohorts investigated (pharmacy, nursing, and medical students) showed similar knowledge on the factors required for estimating absolute CVD risk. However, our findings also showed that the majority (53%) were unable to identify all six risk factors required to estimate an individual’s CVD risk, and a third (32%) being unable to identify TC as an independent risk factor necessary to estimate CVD risk. We acknowledge that the scoring method we used to assess knowledge is simple and the low numbers may not accurately represent all HCSs knowledge of CVD risk factors.

Academic year and months exposed to experiential education did not show correlations with the HCSs’ knowledge scores on CVD risk factors, and there was also no correlation between training scores in the use of CVD risk calculators with students’ knowledge on CVD risk factors. One can speculate that the lack of correlation is a reflection of an overall underutilization of CVD risk assessments in clinical practice which, in turn, decreases the students’ opportunities to observe, use, and understand the clinical value of estimating CVD risk to guide clinical decision-making. Several studies have provided evidence on the poor undertaking of CVDRAM in the part of primary health care professionals (HCPs). In these studies, a CVD risk calculator or CVD prediction rules were used by the minority of primary care doctors and nurses who were surveyed, and there was also limited understanding on the perceived clinical value in assessing CVD risk [21,22,23].

It is possible that this suboptimal adoption of CVD risk assessment in primary care can have a negative impact on student exposure and practice of these skills during experiential education. This could explain the results obtained from pharmacy students who indicated that only a third of their on-campus instruction in CVDRAM is reinforced during experiential education. This may also help explain other results in our study, such as almost a third of medical students, and the majority of nursing students, indicating having received no training on the use of CVD risk calculators, even though training for both groups occurs mostly in the clinical setting.

Overall, students in the three cohorts perceived to be well prepared and confident for the provision of CVDRAM upon graduation. Interestingly, the students’ overall perceived preparedness/confidence scores positively correlated with their training on the use CVD risk calculators, but not with their knowledge score on CVD risk factors. Similar findings have been reported by Wu et al. who surveyed nursing students [24]. In this study, the authors also found a discrepancy between students’ perceived preparedness and confidence and their knowledge levels. These type of findings have been explained as a lack of insight on the part of the students into their own strengths and weaknesses, which may lead them to over or underestimate themselves when self-rating their preparedness and confidence in surveys [25].

An interesting finding was that pharmacy students reported the highest preparedness/confidence scores (4) with the use of the latest ACC/AHA CVDRAM guidelines when compared to medical and nursing students (3 and 2, respectively, *p* = 0.001). As reported in our study, because most of the learning on CVDRAM for medical and nursing students is reported to occur in the practice setting, a possible explanation for these students rating their preparedness and confidence on the use of CVDRAM guidelines lower than pharmacy students may be related to poor guideline adherence in the part of their preceptors while learning in practice. This explanation may be supported by some studies with HCPs. In a survey by Doroodchi et al. of general practitioners and general internists on CVDRAM, the authors found gaps in these HCPs’ management of CVD risks according to guideline recommendations [26]. In another survey by Reiner et al., although most physicians supported the use of guidelines, only half used them and, on average, their knowledge of guidelines was not satisfactory [27].

Poor acceptance or unawareness on the part of the public about CVD risk was the only barrier perceived as major by nursing students. As nursing students spend a considerable amount of time in close contact with patients, they may have more insight into how aware the general public is of CVD and its associated risk factors. This perception by nursing students may indicate their concerns that the observed limited knowledge on the part of the public may pose a significant challenge for the provision of CVDRAM. On the other hand, this finding can also be viewed as an opportunity for student involvement in public education, and to reduce the time constraints often voiced by HCPs as a barrier to conducting risk assessments and to explaining patients the findings as a result of these assessments [28]. Pharmacy and nursing students perceived that lack of support on the part of other HCPs was a moderate barrier compared to medical students who thought of it as a minor barrier. This is similar to the results of a study where professional identity and the low expectations of the pharmacist’s role on the part of the public and among other HCPs were barriers that restricted pharmacists in the provision of comprehensive CVD prevention strategies [29]. Although the differences in the barrier scores for the statement “CVD risk assessment is not an accepted scope of practice within my profession” (0 by medical students, 0.5 by nursing students, and 1 by pharmacy students) were not statistically significant (*p* = 0.82), it is worth commenting that the relative difference between the scores is greater than any of the differences for the other barrier statements. This finding suggests a clear delineation of roles for the medical students who, when compared to nursing and pharmacy students, their role in CVD risk assessment is well-defined and, consequently, it is not viewed as a barrier in the provision of comprehensive CVD prevention strategies [29]. The lack of statistical significance in some of our results is likely to be the result of the low response rate.

The college of Pharmacy in Qatar University, Canadian accredited, has established an IPEC Committee in 2014 to provide guidance and support in implementing IPE within its pharmacy program and other healthcare programs in Qatar and is always exploring innovative IPE approaches [30]. As such and based on the findings of this survey, a recommendation has been made to the College of Pharmacy IPE Committee to initiate an interprofessional approach to deliver a CVDRAM to HCSs in collaboration with other institutions. It is possible that this strategy can strengthen not only the students’ knowledge and skills, but it may also have an impact on the uptake of CVDRAM in clinical practice by their experiential educators. Obviously, such an outcome would need to be tested. There is substantial evidence that IPE provides HCSs with the opportunity to learn with, from, and about each other to improve collaboration and quality of care to patients [31]. It is also an opportunity to learn from each other’s strengths and of each other’s professional roles, and minimize barriers in the provision of collaborative care [32]. 

There are a number of limitations to this study that need to be considered. As with most survey research methodology, a major limitation was the low response rate, which resulted in a low sample size for some of the cohorts. Our study sample was also limited to three colleges and, therefore, it may not be representative of Qatar’s HCSs who are being trained for the provision of CVDRAM. There were also some limitations regarding the administration of the questionnaire, as some HCSs completed the questionnaires online (for medical and nursing students). This may have introduced bias in the knowledge scores as we cannot assure these students did not access the Internet to look for answers. Most HCSs surveyed in this study were interested in secondary or tertiary care practice, which may have introduced bias when responding to questions that are mostly focused in primary care practice. Another limitation is that all of the items included in the preparedness and confidence scale were positive statements without alternating item wording, which may be associated with acquiescent biases. Although, when these scales were tested through the use of “Alpha if Item Deleted”, most revealed very good internal consistency and only three statements would be advisable to be reworded in future research using this questionnaire. 

## 5. Conclusions 

This study revealed that HCSs in Qatar reported similar levels of knowledge and training in CVDRAM, and perceived themselves to be well prepared and confident for the provision of CVDRAM services upon graduation. Although the sample size was small, the results point towards some knowledge and training gaps in CVDRAM among HCSs that are worth assessing further. This information may be particularly useful when reviewing and designing the experiential education curriculum in CVDRAM for HCSs. Future integration of IPE activities in the CVDRAM curricula of HCSs is a strategy worth exploring to facilitate collaborative practice and to decrease some of the associated barriers associated with the provision of CVDRAM services in Qatar.

## Figures and Tables

**Table 1:** Students’ characteristics.

Characteristics	Total *n* = 81 (% of Total)
**Discipline** Pharmacy 3rd 4th Medicine 3rd 4th Nursing 3rd 4th	35 (43.2%) 17 (20.9%) 18 (22.2%) 25 (30.8%) 14 (17.3%) 11 (13.6%) 21 (26%) 13 (16%) 8 (9.9 %)
**Gender** Female Male	67 (82.7%) 14 (17.3%)
**Total durations of Clinical Placements** Less than 1 month 1–3 months 4–6 months More than 6 months	1 (1.2%) 25 (30.7%) 25 (30.7%) 30 (37%)
**Preferred workplace after graduation** Primary care Secondary/Tertiary Academia Other (school, research, surgery related, etc.)	6 (7.4%) 54 (66.7%) 14 (17.3%) 7 (8.6%)

*n* = number of student respondents, 3rd = third year students, 4th = fourth year students.

**Table 2:** Students’ ability to identify the risk factors required for estimating absolute CVD risk.

	Students’ Discipline			*p* Value **
	Pharmacy *n* (%)	Medicine *n* (%)	Nursing *n* (%)	
T	17 (49%)	9 (36%)	12 (57%)	0.29
**Risk factors**				
Age	33 (94%)	25 (100%)	21 (100%)	0.264
Gender	29 (82.8%)	24 (96%)	19 (90.5%)	0.030 *
Blood Pressure	32 (91.4%)	22 (88%)	21 (100%)	0.500
Smoking Status	33 (94.3%)	25 (100%)	21 (100%)	0.264
TC	25 (73.5%)	15 (60%)	15 (71.4%)	0.003 *
HDLC	29 (82.9%)	17 (68%)	18 (85.7%)	0.096

T = number of students able to identify all main six risk factors required for estimating absolute CVD risk, *n* = number of students who identified this risk factor as required for estimating absolute CVD risk, HDLC = high density lipoprotein cholesterol, * Indicates significant differences between the three student cohorts, ** Kruskal Wallis Test.

**Table 3:** Students’ perceived barriers for the provision of cardiovascular disease risk assessment and management.

Statements	Students’ Discipline			*p* Value **
	Medicine Median (IQR)	Nursing Median (IQR)	Pharmacy Median (IQR)	
**Lack of time to perform CVD risk assessments**	3.00(2)	3.00(2)	3.00(2)	0.58
**Lack of adequate space to perform CVD risk assessments**	1.00(3)	3.00(2)	3.00(2)	0.17
**Lack of resources (e.g., access to guidelines, medical health records, laboratory data, etc.)**	1.00(3)	2.50(3)	2.00(3)	0.28
**Lack of support (e.g., other health care providers’ acceptance, hospital managers/directors, government, etc.)**	2.00(3)	3.00(2.25)	3.00(3)	0.01 *
**Lack of confidence and training in CVD risk assessment and management**	3.00(2)	3.00(2.25)	2.00(2)	0.21
**Poor public acceptance or unawareness of importance of estimating CVD risk**	2.00(2)	4.00(2.25)	2.00(3)	0.001 *
**CVD risk assessment is not an accepted scope of practice within my profession**	0.00(2)	0.50(3)	1.00(2)	0.82

IQR = interquartile ranges, CVD = cardiovascular disease, * Indicates significant differences between the three student cohorts, ** Kruskal-Wallis test based on medians.

## Supplementary Materials

The following are available online at www.mdpi.com/2226-4787/5/1/9/s1: Questionnaire: Health care students’ views on their training and role in cardiovascular disease risk assessment and management.
