# Supplementary material for: Perceived Preparedness of Health Care Students for Providing Cardiovascular Disease Risk Assessment and Management
Source: Pharmacy (Basel). 2017 Feb 21;5(1):9. doi: 10.3390/pharmacy5010009 (PMC5419389; doi:10.3390/pharmacy5010009)
Supplement: Supplementary file 1 [file pharmacy-05-00009-s001.pdf]

Health care students' views on their training and role in cardiovascular disease risk assessment and management

Dear participant,

Thank you very much for agreeing to participate in this survey. Completion of the survey is an indication that you have agreed to be a participant of this study. Please be reassured that this survey is confidential. Please complete the survey to the best of your ability and as completely as possible.

Please state your discipline:

- ☐ Medicine
- ☐ Nursing
- ☐ Pharmacy

Current Year of medical/nursing/pharmacy studies. Please Specify___________________

The following abbreviations have been used throughout the questionnaire:

BMI= Body mass index

INR= International Normalized Ratio

BMD= Bone mineral density

CVD = Cardiovascular disease

TC = Total cholesterol

LDL = Low-density lipoprotein

HDL = High-density lipoprotein

TG = Triglycerides

ACC/AHA= American College of Cardiology/American Heart Association

---

PLEASE PROCEED TO THE QUESTIONNAIRE

SECTION 1:

1. Gender:
- ☐ Male
- ☐ Female

2. Indicate the total duration of your experiential placements (clinical rotations) thus far:
- ☐ None
- ☐ Less than 1 month
- ☐ 1-3 months
- ☐ 4-6 months
- ☐ More than 6 months

3. Where would you like to work after graduation?
- ☐ Primary care (e.g. primary health care clinics, outpatient clinics, community pharmacies)
- ☐ Secondary/Tertiary care (e.g. hospitals)
- ☐ Academia (e.g. universities)
- ☐ Other *(please specify):* ______________

SECTION 2:

4. From the following list of assessments, please indicate the items that you received hands-on training during your undergraduate education and if you had the opportunity to use these skills during your clinical training (e.g. placements or rotations).

✓ **Tick all that apply.**

	On Campus (in class or labs)	In clinical training	Cannot recall	No training provided
Blood pressure measurement				
Pulse measurement				
Heart sounds				
Blood glucose testing (e.g. capillary fingerprick)				
Urine glucose testing (dipstick)				
Blood cholesterol testing (e.g. capillary fingerprick)				
BMI measurement				
Waist circumference measurement				
INR testing				
BMD measurement				
Used point-of-care instruments (e.g: glucose/blood pressure device)				
Used CVD risk calculators (e.g: Framingham, QRISK)				

PLEASE PROCEED TO QUESTION 5 OF THE QUESTIONNAIRE ON THE BACK OF THIS PAGE

5. From the following list, please indicate which of the following are needed and which are not, to estimate an individual's CVD risk.

	No	Yes	Do not know
Race/ethnicity			
Age			
Gender			
Smoking status			
Family history of CVD			
Presence of diabetes			
Presence of chronic kidney disease			
Taking medications for blood pressure			
BMD			
Blood pressure			
BMI			
Waist circumference			
Heart rate			
Blood glucose level			
C-reactive protein			
Creatinine clearance			
INR			
LDL-cholesterol level			
TC level			
HDL-cholesterol level			
TC:HDL ratio			
TG level			

PLEASE PROCEED TO QUESTION 6 OF THE QUESTIONNAIRE ON THE NEXT PAGE

SECTION 3:

6. **Please indicate your level of agreement or disagreement with the following statements in regards to your perceived preparedness in CVD risk assessment and management.**

Rating: 1 = strongly disagree; 2 = disagree; 3 = neutral; 4 = agree; 5 = strongly agree

Student's opinion regarding:	1	2	3	4	5
**Overall,** I feel my training in my discipline (Pharmacy/Medical/Nursing) has provided me with the physical assessment skills necessary to screen patients for CVD.					
I feel confident identifying individuals that may be at risk of developing CVD.					
I feel confident interviewing patients to identify the presence of CVD risk factors.					
I feel confident using CVD risk calculators that estimate CVD risk (eg. 10-year ACC/AHA CVD risk calculator).					
**Overall**, I feel my training has provided me with the knowledge base required to screen patients for CVD.					
Iam aware of and understand the recommendations provided by the 2013 ACC/AHA Guidelines on CVD risk assessment and management.					
I feel confident interpreting findings derived from performing a CVD risk assessment.					
I feel confident recommending strategies to reduce CVD risk based on findings derived from performing a CVD risk assessment.					
I feel confident counseling patients on CVD risk reduction strategies.					
I feel confident recommending pharmacologicaltreatments to reduce CVD risk.					
I feel confident recommending non-pharmacological treatments to reduce CVD risk.					
I feel confident monitoring patients who are on treatment for reducing their CVD risk.					
**Overall**, I feel well prepared to provide CVD risk assessment and management services for patients when I graduate.					

PLEASE PROCEED TO QUESTION 7 (AND THE LAST ONE) ON THE BACK OF THIS PAGE

SECTION 4:

7. **From your observations during your clinical training and experiential placements, indicate which of the following barriers you perceive your profession is facing for being involved in assessing patients at risk of CVD.**

   Rating: 0 = not a barrier, 5 = major barrier

Perceived Barriers	0	1	2	3	4	5
Lack of time to perform CVD risk assessments						
Lack of adequate space to perform CVD risk assessments						
Lack of resources (e.g. access to guidelines, medical health records, laboratorydata,etc.)						
Lack of support (e.g. other health care providers' acceptance, hospital managers/directors, government, etc.)						
Lack of confidence and training in CVD risk assessment and management						
Poor public acceptance or unawareness of importance of estimating CVD risk						
CVD risk assessment is not an accepted scope of practice within my profession						

THANK YOU FOR COMPLETING THE SURVEY
